# Nimodipine Promotes Functional Recovery After Spinal Cord Injury in Rats

**DOI:** 10.3389/fphar.2021.733420

**Published:** 2021-09-13

**Authors:** Fangliang Guo, Xiaolong Zheng, Ziyu He, Ruoying Zhang, Song Zhang, Minghuan Wang, Hong Chen, Wei Wang

**Affiliations:** ^1^ Department of Neurology, Tongji Hospital, Tongji Medical College, Huazhong University of Science and Technology, Wuhan, China; ^2^ Department of Rehabilitation, Tongji Hospital, Tongji Medical College, Huazhong University of Science and Technology, Wuhan, China

**Keywords:** nimodipine, spinal cord injury, contusion, neuropathic pain, spasticity

## Abstract

Spinal cord injury (SCI) is a devastating condition that results in severe motor, sensory, and autonomic dysfunction. The L-/T-type calcium channel blocker nimodipine (NMD) exerts a protective effect on neuronal injury; however, the protective effects of long-term administration of NMD in subjects with SCI remain unknown. Thus, the aim of this study was to evaluate the role of long-term treatment with NMD on a clinically relevant SCI model. Female rats with SCI induced by 25 mm contusion were subcutaneously injected with vehicle or 10 mg/kg NMD daily for six consecutive weeks. We monitored the motor score, hind limb grip strength, pain-related behaviors, and bladder function in this study to assess the efficacy of NMD in rats with SCI. Rats treated with NMD showed improvements in locomotion, pain-related behaviors, and spasticity-like symptoms, but not in open-field spontaneous activity, hind limb grip strength or bladder function. SCI lesion areas and perilesional neuronal numbers, gliosis and calcitonin gene-related peptide (CGRP^+^) fiber sprouting in the lumbar spinal cord and the expression of K^+^–Cl^−^ cotransporter 2 (KCC2) on lumbar motor neurons were also observed to further explore the possible protective mechanisms of NMD. NMD-treated rats showed greater tissue preservation with reduced lesion areas and increased perilesional neuronal sparing. NMD-treated rats also showed improvements in gliosis, CGRP^+^ fiber sprouting in the lumbar spinal cord, and KCC2 expression in lumbar motor neurons. Together, these results indicate that long-term treatment with NMD improves functional recovery after SCI, which may provide a potential therapeutic strategy for the treatment of SCI.

## Introduction

Spinal cord injury (SCI) is a devastating condition that leads to severe problems involving impaired motor, sensory, and autonomic functions (Ahuja et al., 2017). A substantial number of people with SCI (70–80%) develop neuropathic pain or spasticity over time (Finnerup et al., 2014; Andresen et al., 2016). Neuropathic pain and spasticity impair motor recovery, worsen quality of life, and even lead to early mortality (Krause et al., 2009). The current management of SCI includes spinal immobilization, surgical decompression, pharmacological therapies and rehabilitation (Ahuja et al., 2017).

The initial traumatic impact results in a sustained cascade of secondary injury, including apoptosis, ischemia, vasospasm, disruption of ionic homeostasis and inflammation, which propagate the damage and worsen the function (Tator and Fehlings, 1991; Ahuja et al., 2017). Thus, approaches that mitigate secondary injury after SCI may lead to better functional improvement (Silva et al., 2014; Ahuja et al., 2016). Nimodipine (NMD) is an L-/T-type calcium channel blocker and has been approved for the treatment of subarachnoid hemorrhage (SAH) by the U.S. Food and Drug Administration (Macdonald and Schweizer, 2017). The effects of NMD mainly include central nervous system (CNS)-specific vasodilation and blockade of the flux of extracellular calcium through L-/T-type calcium channels (Gurkoff et al., 2013; Carlson et al., 2020). Recent studies have shown that it also prevents the development of spasticity after SCI and reduces CNS inflammatory responses (Schampel et al., 2017; Marcantoni et al., 2020; Zamora et al., 2020). NMD has been tested in preclinical (Tator et al., 2012) and clinical SCI research (Pointillart et al., 2000); however, its efficiency remains elusive. Perhaps one of the reasons may be the short duration of the treatment, the longest of which is 1 week (Pointillart et al., 1993; Ross et al., 1993; Pointillart et al., 2000). In addition, the sample size in the clinical trial might have been too small to detect a significant clinical effect (Pointillart et al., 2000). Based on these observations, long-term administration of NMD after SCI may improve function. Thus, this study aims to evaluate the efficiency of long-term therapy with NMD in a clinically relevant SCI model.

Here, we observed that long-term administration of NMD showed improvements in locomotor function, pain-related behaviors, and spasticity-like symptoms, but not in bladder function after a moderate-to-severe spinal cord contusion injury. Furthermore, the possible mechanisms by which NMD exert its actions were explored by assessing the lesion area, peri-lesional neuronal numbers, gliosis and calcitonin gene-related peptide (CGRP^+^) fiber sprouting in the lumbar spinal cord and the expression of K^+^–Cl^−^ co-transporter 2 (KCC2) on lumbar motor neurons.

## Materials and Methods

### Ethics Statement and Animals

All animal experiments were performed in compliance with protocols approved by the Institutional Animal Care and Use Committee at Tongji Hospital, Tongji Medical College of Huazhong University of Science and Technology. We made all efforts to minimize animal suffering and the number of animals used. Adult female Sprague-Dawley rats of specific pathogen-free grade were purchased from Beijing HFK BIOSCIENCE CO. LTD (Beijing, China). All animals were acclimated and trained for the baseline behavioral assessment prior to SCI. Rats were provided *ad libitum* access to food and water and housed in groups of two rats per cage on 12 h day/night cycles with bedding changed frequently.

### Spinal Contusion Injury and Animal Care

Surgeries were conducted on adult female rats (260–300 g) at the age of 9–11 weeks. The procedure was similar to that described previously (Gong et al., 2021). Briefly, rats were anesthetized with isoflurane (induction at 4%, maintenance at 2%) in oxygen. The back fur was shaved, and the bladder was evacuated before surgery. Both eyes were covered with eye ointment to prevent xerophthalmia during anesthesia. Aseptic conditions were used for all surgical procedures. After skin preparation and disinfection, the skin overlying the vertebral column was incised, and the muscles were detached from the vertebra. A laminectomy was then performed at thoracic level 10 (T10) and the caudal portion of T9 to expose the dorsal spinal cord surface. We stabilized the spinal cord in a small animal spinal holder (RWD, Inc., China) by clamping the T8 and T11 vertebrae with stabilizing forceps. Spinal contusion injury was performed with a metal rod (10-g weight) dropped from a height of 25 mm onto the exposed dorsal surface of the spinal cord using a modified NYU impactor (Li et al., 2020). After spinal contusion, the wound was rinsed with normal saline and sutured in layers. Rats were placed on a 37°C heating pad throughout the operation until arousal. After SCI, rats received subcutaneous (sc) analgesic treatment (carprofen, Pfizer Inc., United States, 5 mg/kg) for 3 days. Saline (5 ml, sc) and ampicillin (North China Pharmaceutical Company Ltd., China, 100 mg/kg, sc) were administered daily for 7 days to prevent dehydration and infection, respectively. Bladders were expressed manually three times daily until spontaneous voiding was restored. The body weight (BW) of each rat was measured every 4 days until 6 weeks post-SCI and then weekly until 12 weeks.

### Pharmacological Treatment and Animal Groups

**FIGURE 1:**
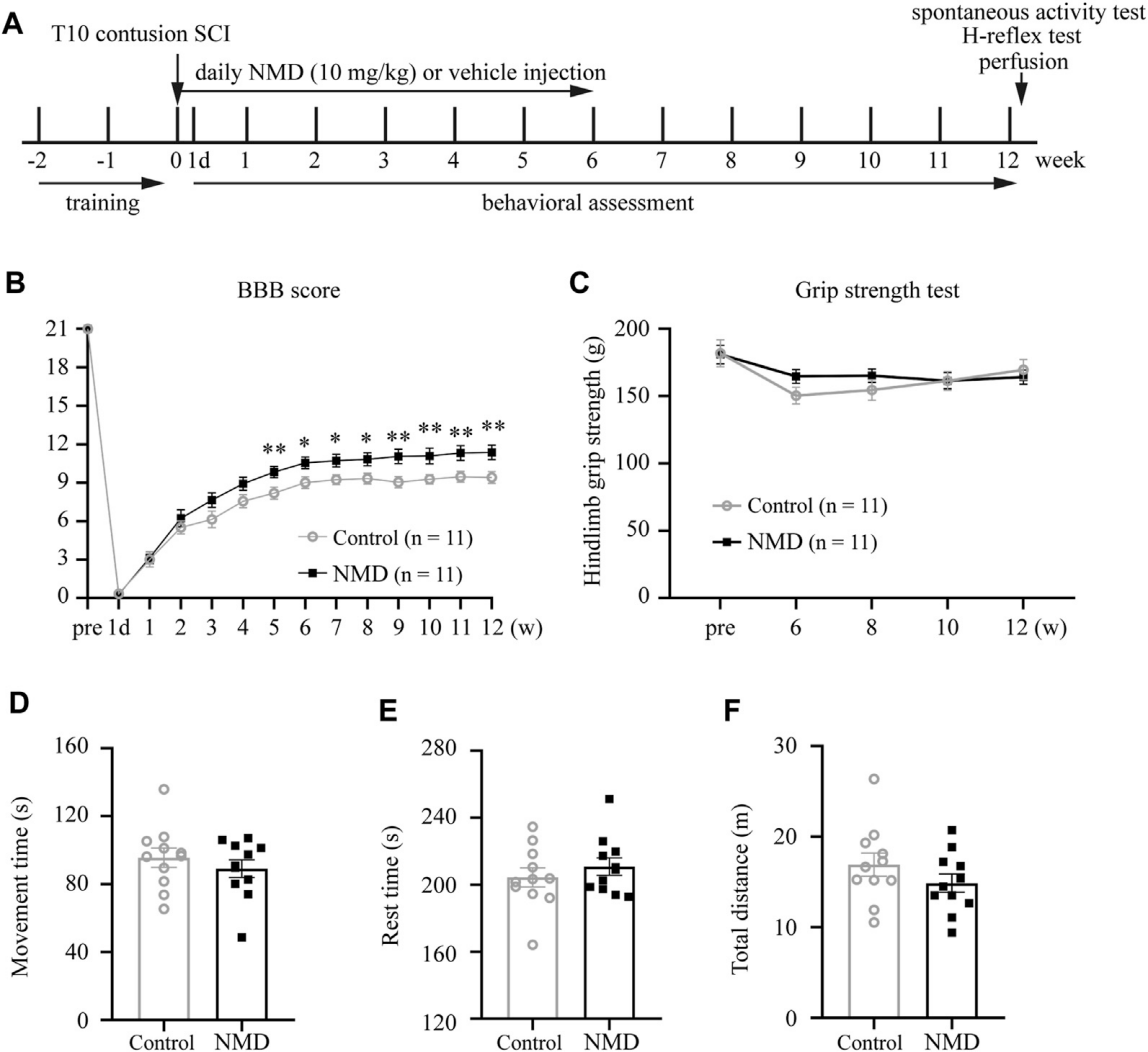
Summary of the experimental design and functional assessments of NMD and control rats following SCI. **(A)** Schematic illustrating the chronological order of experiments for SCI, drug administration, and functional assessments. **(B)** Significant improvements in Basso, Beattie, and Bresnahan (BBB) scores were observed in NMD-treated rats (*n* = 11 in each group). Generalized estimating equation, *p* = 0.008 (week 5), 0.013 (week 6), 0.012 (week 7), 0.016 (week 8), 0.004 (week 9), 0.008 (week 10), 0.006 (week 11), and 0.005 (week 12). *p < 0.05, **p < 0.01. **(C)** Hind limb grip strength test (*n* = 11 rats in each group). Two-way repeated-measures ANOVA, *p* > 0.05, F (1, 20) = 0.284. **(D–F)** Open-field spontaneous activity test (*n* = 11 rats in each group). Unpaired t-test, *p* > 0.05.

NMD (MCE; catalog no. HY-B0265/CS-2253) for subcutaneous injection was used as described in previous studies (10 mg/kg) (Schampel et al., 2017; Marcantoni et al., 2020). NMD was dissolved in the vehicle solution, which consisted of 10% dimethyl sulfoxide (Sigma; catalog no. 472301), 5% ethanol, 40% polyethylene glycol (MCE; catalog no. HY-Y0873), and normal saline. Because NMD is very sensitive to light, all steps, including stock preparation and administration, were performed in the dark. SCI rats were randomly allocated to two groups: a long-term NMD (*n* = 12) group and a control group (vehicle, *n* = 12). Starting 1 h after injury, NMD was subcutaneously injected daily for 6 weeks. The vehicle solution without NMD was injected into the control animals using the same protocol. One rat in the control group was excluded because its Basso, Beattie, and Bresnahan (BBB) score was 20 at 1 day after SCI, which placed it at >2 s.d. above the mean. One rat in the NMD group was sacrificed before the endpoint because it presented a greater than 20% decrease in BW on day 14 post-SCI. A summary of the experimental design is shown in Figure 1A.

### Functional Assessments

Rats were trained on the behavioral tests before injury. Functional performance was assessed using several measures: 1) Basso, Beattie, and Bresnahan open-field locomotor score for an assessment of hind limb function, 2) grip strength test of hind-limb force, 3) Von Frey test for mechanical allodynia, 4) Hargreaves test for thermal hyperalgesia, 5) open-field spontaneous activity test for mobility, and 6) rate-dependent depression (RDD) of H-reflex for spasticity-like symptoms. Detailed descriptions of these tests are provided below. All outcome measures were assessed by two expert, independent, and blinded observers.

#### Basso, Beattie, and Bresnahan Locomotor Score

Locomotor function was evaluated using the BBB open-field 21-point locomotion rating scale (Basso et al., 1995). Rats were placed individually in an open field, and hind limb motor function was video-recorded and scored for 4 min. Rats were tested before injury, on day 1 after SCI and weekly thereafter until week 12.

#### Grip Strength Test

Hind limb grip strength was assessed using a grip strength meter (IITC Life Sciences, Inc., Woodland, CA, United States) as previously described (Kramer et al., 2009; Huang et al., 2013). Rats were acclimated to the hind limb T-bar for three consecutive days prior to data collection. The rat was held upright by the scruff of the neck, and it was lowered over the T-bar with its abdomen facing the T-bar. Once it grasped the bar, it was pulled backward with the tail in a horizontal plane. At the moment of release from the bar, the grip force was recorded from the display (grams). Then, the rat was returned to its cage, and the meter was tared (zero). This procedure was repeated with the next subject in the same cage. The test was repeated five times for each hindpaw. This protocol was repeated for two consecutive days, and the force measurement for each paw was averaged.

#### Von Frey Test

The mechanical withdrawal threshold was measured with an electronic Von Frey apparatus (Electronic Von Frey Anesthesiometer 2450, IITC Life Sciences, Inc., Woodland, CA, United States) using a previously described procedure (Parada et al., 2003; Gong et al., 2021). The application of the Von Frey test requires weight support, as evidenced by a score of 9 during BBB locomotor scoring (Detloff et al., 2010; Fouad et al., 2020). Two rats in the control group and one in the NMD group did not recover weight support steps at 6 weeks post-SCI, which reduced the number of animals to *n* = 9 in the control group and *n* = 10 in the NMD group. Rats were placed in Plexiglas chambers on a wire mesh floor in a quiet room for 45 min of acclimation starting 3 days before testing. On the day of testing, rats were allowed 15 min to acclimate to the environment. A tilted mirror was placed under the mesh floor to provide a clear view of the hindpaw. During this adaptation period, the hindpaws were stimulated at the center several times with a gradual increase in pressure. The test consisted of inducing a hindpaw withdrawal response with a rigid tip (diameter = 1 mm) attached to the recording apparatus. When the hindpaw was withdrawn, the stimulus was automatically discontinued, and the value was recorded and displayed by the device automatically to reflect the pain threshold. The maximum force applied was 80 g. The procedure was repeated five times with a minimum 1 min interval between stimuli. Values from each pair of hindpaws were averaged at each time point.

#### Hargreaves Test

Thermal hyperalgesia was measured using a plantar heat testing device (IITC Life Sciences, Inc., Woodland, CA, United States) as described previously (Walker et al., 2019). Briefly, rats were placed in Plexiglas chambers on a transparent glass plate for 45 min of acclimation starting 3 days before testing. On the day of testing, rats were allowed 15 min to acclimate to the environment. The hindpaw was stimulated at the center with a light beam (intensity of 30), and the latency of paw withdrawal was recorded in seconds. The cutoff time was set to 20 s to avoid paw injury. The procedure was repeated five times with a minimum 1 min interval for each hindpaw. The hindpaw withdrawal latency was recorded as an average of the 10 trials per animal at each time point.

#### Open-Field Spontaneous Activity Test

At 12 weeks post-SCI, the open-field spontaneous activity test was performed using previously described procedures (Zuo et al., 2020). Briefly, each rat was placed individually in an open-field box (100 cm × 100 cm × 35 cm) for 5 min. The test parameters, including total distance traveled, rest time, and movement time, were recorded automatically using an ANY-MAZE video tracking system (Stoelting, United States).

#### Hoffmann Reflex Testing

At 12 weeks post-SCI, the H-reflex was recorded by a Dantec Keypoint EMG Workstation (Natus, United States) as described previously (Boulenguez et al., 2010; Gong et al., 2021). Briefly, the rats were anesthetized with 1.5% isoflurane in oxygen. The cathode was inserted above the ankle joint to stimulate the tibial nerve, and the anode was placed below the ankle approximately 1 cm from the cathode. The recording electrode was inserted into the same side of the interosseous muscles subcutaneously between the fourth and fifth metatarsals, and a reference electrode was inserted subcutaneously in the third digit of the hindpaw. In addition, a ground electrode was attached to the surface of the plantar.

H-waves and M-waves were first recorded at 0.1 Hz with a 0.2 ms pulse duration and a gradual increase in stimulus intensities (0.1 mA increments) to determine the maximal amplitudes of the H-wave (Hmax) and M-wave (Mmax).

The rate-dependent depression (RDD) of the H-reflex was estimated as described previously (Boulenguez et al., 2010; Bilchak et al., 2021). Briefly, the stimulation intensity that evoked the Hmax response was used for a series of 20 consecutive stimulations at 0.1, 0.5, 1, 2, and 5 Hz. Then, the 0.1 Hz series was repeated to verify that the M-wave amplitude was still within 95% of the value of the initial trial. The first three responses in a stimulation series were discarded to obtain reflex stabilization, and the peak-to-peak amplitude of the remaining 17 responses was averaged for each animal and frequency. Change in the M-wave and H-wave at each stimulus frequency were calculated as a percentage of the response measured at 0.1 Hz.

#### Bladder Function

Following spinal cord injury, deficits in bladder function are manifested by an inability to empty the bladder, which can be quantified by measuring the weight of urine retained in the bladder. Expressed urine was weighed once weekly during routine bladder expression in the morning to assess urine retention (Sharp et al., 2014). In addition, spontaneous voiding ability was monitored daily in the morning when the bladder was at its fullest. If the bladder was not distended and urine was not expelled during manual bladder expression in the morning and this phenomenon occurred for three consecutive days, the animal was considered to have achieved spontaneous voiding ability (Mothe et al., 2020).

### Histology and Immunohistochemistry

#### Tissue Preparation

Rats were deeply anesthetized and transcardially perfused with ice-cold 0.9% saline followed by 4% paraformaldehyde (PFA) at 12 weeks post-SCI after behavioral assessments. Dissected spinal cords were postfixed in 4% PFA overnight and then immersed in a 30% sucrose solution 3–5 days before embedding and freezing in OCT (SAKURA, United States). Horizontal sections (1.5-cm-long) of the spinal cords containing the lesion site and cross sections of the lumbar enlargement (L4–L5) were sectioned on a cryostat (Leica CM1950, Germany). Serial horizontal sections were collected spanning the dorsal to ventral axis for lesion analysis or immunostaining.

#### Lesion Analysis

For the lesion analysis, 20 µm horizontal sections (120 µm apart) were cut and placed on coated glass slides. Then, sections were stained with hematoxylin and eosin (HE) and imaged with a digital pathology section scanning system (Ocus, Grundium, Finland). The lesion area and the total spinal cord area of the 1.5 cm length section were manually traced using Fiji software (NIH, Bethesda, MD) by an investigator blinded to the group information. Any septae or fibrous bands of tissue within the cavities were counted as part of the lesion. The total lesion volume (lesion volume) and total spinal cord volume (total volume) were calculated using the Cavalieri method (Fan et al., 2013; Mothe et al., 2020). This method is a summation of the measured area of each section multiplied by the intersection distance. The percent lesion area was calculated using the following equation: % Lesion Area = lesion volume/total volume × 100%.

#### Immunofluorescence Staining

For immunostaining, 40 µm free floating sections were cut and stored in PBS at 4°C. Tissue sections were blocked with 5% normal donkey serum (Jackson ImmunoResearch Labs Cat# 017-000-121, RRID: AB_2337258) in PBS containing 0.3% Triton X-100 for 1 hour and then incubated with primary antibodies diluted in blocking buffer overnight at 4°C, followed by an incubation with secondary antibodies for 1 h at room temperature after three washes for 10 min each. The primary antibodies used were chicken anti-GFAP (1:1000, Abcam Cat# ab4674, RRID: AB_304558), rabbit anti-Iba1 (1:500, FUJIFILM Wako Shibayagi Cat# 019-19741, RRID: AB_839504), guinea pig anti-NeuN (1:1000, Millipore Cat# ABN90, RRID: AB_11205592), rabbit anti-CGRP (1:1000, Peninsula Laboratories Cat# T-4032, RRID: AB_518147), rabbit anti-KCC2 (1:500, Millipore Cat# 07-432, RRID: AB_310611), and goat anti-ChAT (1:100, Millipore Cat# AB144P, RRID: AB_2079751). The following secondary antibodies were diluted 1:1000 before use: Alexa Fluor 488-labeled donkey anti-rabbit antibody (Thermo Fisher Scientific Cat# A-21206, RRID: AB_2535792), Cy3-labeled donkey anti-chicken antibody (Jackson ImmunoResearch Labs Cat# 703-165-155, RRID: AB_2340363), Cy3-labeled donkey anti-goat antibody (Jackson ImmunoResearch Labs Cat# 705-165-003, RRID: AB_2340411), and Alexa Fluor 647-labeled donkey anti-guinea pig antibody (Jackson ImmunoResearch Labs Cat# 706-605-148, RRID:AB_2340476). After staining, the tissue sections were incubated with Hoechst 33258 for 15 min. Then, the sections were washed with PBS for 30 min, mounted on glass microscope slides, dried, and cover-slipped before imaging.

#### Imaging and Quantification

Immunofluorescence images were captured with identical settings and exposure times using an FV-3000 confocal microscope (Olympus, Japan). Investigators blinded to the group information performed the quantification using Fiji software. For NeuN quantification, the sampled region extended from 4 mm rostral to 4 mm caudal to the lesion epicenter. NeuN^+^ cells were quantified within the epicenter of the sampled region in two sections (160 µm apart) from each animal to avoid double counting of cells (Mothe et al., 2020). Cross sections (three sections per animal) from the lumbar enlargement were used to quantify GFAP and Iba1 labeling. The percentage of GFAP^+^ area, the GFAP or Iba1 intensity, and the number of positively labeled Iba1^+^ cells were measured in a region of interest (ROI: a square area with a side length of 250 μm) located within the dorsal horn (Gwak et al., 2012). The measurement was the average of the left and right dorsal horn per animal. For the calcitonin gene-related peptide (CGRP^+^) axon analysis, cross sections (three sections per animal) from the lumbar enlargement were immunostained for CGRP. Using the Atlas of the Rat Spinal Cord (Watson et al., 2009), spinal lamina I-II and III-V were outlined on each image using the freehand selection tool in Fiji (Brennan et al., 2021). The area of CGRP^+^ staining in each ROI was selected using the threshold tool in Fiji and recorded as a percentage of the lamina area. The area of CGRP^+^ axons in lamina I-II and III-V was reported the average of the left and right dorsal horns. For K^+^–Cl^−^ cotransporter 2 (KCC2) quantification, thirty-six motor neurons from the lumbar enlargement were averaged in each group (six motor neurons for each animal, *n* = 6 per group) and were identified with choline acetyltransferase (ChAT^+^) labeling, a typical large size, and a location within the ventral horn. The fluorescence intensity of KCC2 immunolabeling on the motoneuronal membrane was measured using Fiji software and the average integrated area of the density curve was obtained by drawing three lines across each motoneuron (yielding six data points per cell) (Boulenguez et al., 2010; Liabeuf et al., 2017; Bilchak et al., 2021). Mean pixel intensities for membranes were normalized to intensities in the cytoplasm of each motor neuron to examine the amount of KCC2 expressed in the membrane compared to the cytosol (Boulenguez et al., 2010).

### Bladder Tissue Processing and Quantification

Bladder tissue was prepared as described previously (Fandel et al., 2016; Mothe et al., 2020). Prior to perfusion, bladders were removed, and fixed with 4% PFA overnight, and cryoprotected in 30% sucrose at 4°C for 3 days. Then, bladders were weighed and photographed with a ruler. The circumference of each bladder was measured using Fiji software and averaged for each group. For the quantification of the thickness of the detrusor muscle, the bladders were bisected longitudinally from the dome to the neck and embedded in OCT compound. Then, 10-μm-thick frozen sections were cut and stained with HE, and six consecutive bladder sections per rat were analyzed. Images were captured using a BX-51 microscope (Olympus, Japan). For each bladder, the thickness of the detrusor muscle was measured using the Fiji software and averaged for each animal.

### Statistical Analysis

All statistical analyses were performed using GraphPad Prism 8.0 software or SPSS Statistics 18. Sample sizes were determined based on previous publications assessing the treatment of SCI using pharmacological approaches (Ryu et al., 2018; Sandner et al., 2018; Mothe et al., 2020; Yamazaki et al., 2020). We first performed the Shapiro-Wilk normality test on each dataset. Nonparametric tests were used for data with a non-normal distribution. Two-tailed unpaired t-tests, generalized estimating equation and two-way repeated-measures ANOVA followed by Fisher’s LSD or Bonferroni’s post hoc comparison were used. Statistical significance was set to *p* < 0.05. Data are presented as the means ± SEM.

## Results

### Long-Term Treatment With Nimodipine Improves Motor Function

Previous studies indicate that NMD exerts a protective effect on neuronal injury and prevents the development of spasticity after SCI (Tator et al., 2012; Marcantoni et al., 2020). We used a clinically relevant SCI model and administered vehicle or NMD (10 mg/kg) daily for 6 weeks after SCI to determine whether long-term administration of NMD promotes functional recovery after SCI (Figure 1A). Locomotor function was evaluated before injury, on day 1 after injury and weekly thereafter until week 12 using the BBB locomotor score. Animals that received long-term NMD administration showed a significant increase in the BBB score beginning at 5 weeks after SCI compared to control group. At 12 weeks after SCI, the control rats showed only weight supported steps with no forelimb-hindlimb (FL-HL) coordination (mean score 9.41 ± 0.46), whereas, the NMD-treated rats displayed occasional to consistent weight supported plantar steps (mean score 11.36 ± 0.56). Occasional to frequent FL-HL coordination was also observed in some NMD-treated rats [generalized estimating equation, post hoc by LSD test; Figure 1B]. Long-term administration of calcium channel blockers may lead to muscle weakness (Zamponi et al., 2015); thus, we monitored the hind limb grip strength prior to injury and from week 6 to week 12 post-SCI. The grip strength tests showed no significant difference between controls or NMD-treated animals [two-way repeated-measures ANOVA, F (1, 20) = 0.284, *p* > 0.05; Figure 1C]. A recent report has shown that mice treated with gabapentin (GBP, a calcium channel blocker) rest in an open field more than controls (Sun et al., 2020). Thus, we recorded spontaneous open-field activity at 12 weeks after SCI. No differences were observed in the movement time [unpaired t-test, t (20) = 0.8325, *p* > 0.05; Figure 1D], rest time [unpaired t-test, t (20) = 0.8311, *p* > 0.05; Figure 1E] or total distance traveled between groups [unpaired t-test, t (20) = 1.251, *p* > 0.05; Figure 1F]. Based on these results, long-term treatment with NMD after SCI improves locomotor function without affecting hind limb grip strength and spontaneous open-field activity, suggesting that NMD is beneficial for motor recovery.

No animals died during this study, suggesting that long-term NMD administration (10 mg/kg) does not lead to early mortality. In addition, both the NMD and control groups gained weight over the course of the experiment [F (1.624, 32.49) = 30.26, *p* < 0.0001; Supplementary Figure S1], and no differences were observed in weight between groups using two-way repeated-measures ANOVA [F (1, 20) = 0.1761, *p* > 0.05]. Thus, long-term treatment with NMD does not affect the survival rate and BW.

### Long-Term Treatment With Nimodipine Attenuates Pain-Related Behaviors

**FIGURE 2:**
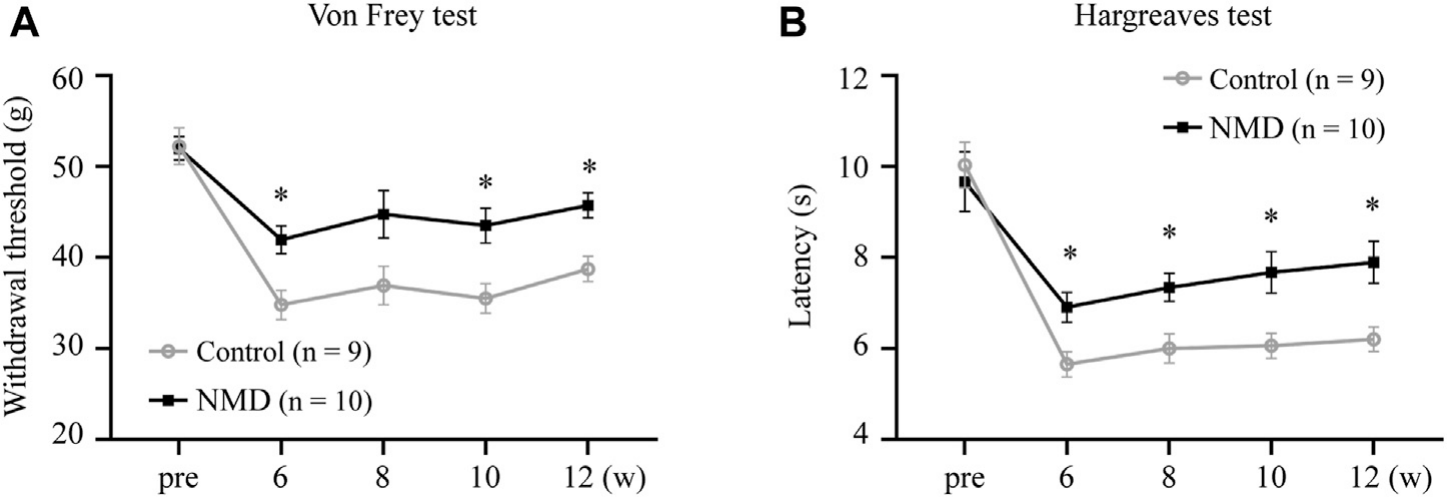
Long-term treatment with NMD attenuates pain related behaviors. **(A)** Graph showing the withdrawal threshold of hind limbs from 6 to 12 weeks (NMD, *n* = 10; controls, *n* = 9). Two-way repeated-measures ANOVA, *p* < 0.05, F (1, 17) = 8.112; post hoc Bonferroni analysis *p < 0.05 at weeks 6, 10, and 12. *p* = 0.0249 (week 6), 0.0282 (week 10), and 0.0117 (week 12). **(B)** Graph showing the withdrawal latency of hind limbs from 6 to 12 weeks (NMD, *n* = 10; controls, *n* = 9). Two-way repeated-measures ANOVA, *p* < 0.05, F (1, 17) = 7.109; post hoc Bonferroni analysis *p < 0.05 at weeks 6, 8, 10, and 12. *p* = 0.0475 (week 6), 0.0392 (week 8), 0.0412 (week 10), and 0.0337 (week 12).

SCI results in neuropathic pain, which is frequently severe and disabling (Finnerup, 2013; Finnerup et al., 2014). Animals were tested for sensory behaviors before injury, beginning at week 6 after SCI and then every 2 weeks thereafter to evaluate whether long-term treatment with NMD ameliorates pain-related behaviors. Our results revealed that NMD-treated rats had significantly higher withdrawal thresholds beginning at 6 weeks after SCI compared to the control group, and the withdrawal threshold was 45.73 ± 1.38 g at week 12 in the NMD group, which was 1.18 times higher than that in the control group [two-way repeated-measures ANOVA, Bonferroni’s post hoc test, *p* = 0.0249 (week 6), 0.0282 (week 10), and 0.0117 (week 12); Figure 2A]. In addition, NMD-treated rats showed a significantly longer latency of paw withdrawal compared to control group, and the latency was 7.89 ± 0.46 at week 12 in NMD group, which was 1.27 times longer than control group [two-way repeated-measures ANOVA, Bonferroni’s post hoc test, *p* = 0.0475 (week 6), 0.0392 (week 8), 0.0412 (week 10), and 0.0337 (week 12); Figure 2B]. Therefore, long-term treatment with NMD after SCI attenuates pain-related behaviors.

### Long-Term Treatment With Nimodipine Alleviates Spasticity-like Symptoms

**FIGURE 3:**
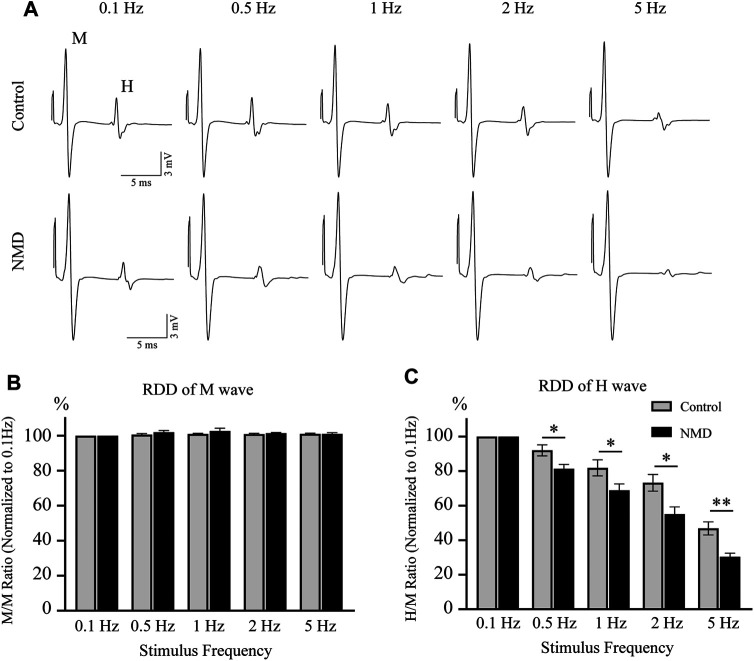
Long-term treatment with NMD alleviates spasticity-like symptoms. **(A)** Representative traces of M and H waveforms upon stimulation at 0.1, 0.5, 1, 2 or 5 Hz. **(B)** The RDD of the M wave in each group is shown (*n* = 11 rats in each group). Two-way repeated-measures ANOVA, *p* > 0.05, F (1, 20) = 1.513. **(C)** Graphs present the RDD of the H wave (*n* = 11 rats in each group). Two-way repeated-measures ANOVA, *p* < 0.01, F (1, 20) = 12.04; post hoc Fisher’s test, *p < 0.05 at 0.5, 1, and 2 Hz, **p < 0.01 at 5 Hz. *p* = 0.0163 (0.5 Hz), 0.0412 (1 Hz), 0.0102 (2 Hz), and 0.0016 (5 Hz).

In both normal humans and rats, the amplitude of the H-reflex is attenuated by repeated activations at frequencies higher than 0.1 Hz (Thompson et al., 1992; Hultborn et al., 1996). Rate-dependent depression (RDD) is reduced in individuals with SCI, and this effect is a reliable indicator of spasticity (Grey et al., 2008; Boulenguez et al., 2010; Gong et al., 2021). We tested the RDD of the H-reflex at 12 weeks after SCI to assess whether long-term treatment with NMD alleviated spasticity-like symptoms. The improvement in RDD observed in NMD-treated animals was characterized by the presence of clear depression when the stimulation frequency increased, while the effect of depression was less obvious on control animals (Figure 3A). As shown in Figure 3B, the M-wave remained unchanged after different stimulation frequencies were applied. Figure 3C shows the average RDD of the H-wave for each group at each frequency as a percentage of the response at 0.1 Hz. A two-way repeated-measures ANOVA revealed statistically significant differences across stimulation frequencies [F (2.279, 45.57) = 161.6, *p* < 0.001] and across experimental groups [F (1, 20) = 12.04, *p* < 0.01] with a significant interaction between frequency and groups [F (4, 80) = 3.766, *p* < 0.01]. Post hoc comparisons suggested that the normalized amplitude of the H-reflex was significantly lower in the NMD group than in the control group at 0.5 Hz (81.4 ± 2.42 vs. 92.01 ± 3.21) (*p* = 0.0163), 1 Hz (68.9 ± 3.63 vs. 81.85 ± 4.66) (*p* = 0.0412), 2 Hz (55.12 ± 4.16 vs. 73.26 ± 4.83) (*p* = 0.0102), and 5 Hz (30.45 ± 2 vs. 46.85 ± 3.78) (*p* = 0.0016) (two-way repeated-measures ANOVA, Fisher’s post hoc test; Figure 3C). Based on these results, long-term treatment with NMD after SCI alleviates spasticity-like symptoms.

### Long-Term Treatment With Nimodipine Enhances Tissue Preservation and Perilesional Neuronal Sparing

**FIGURE 4:**
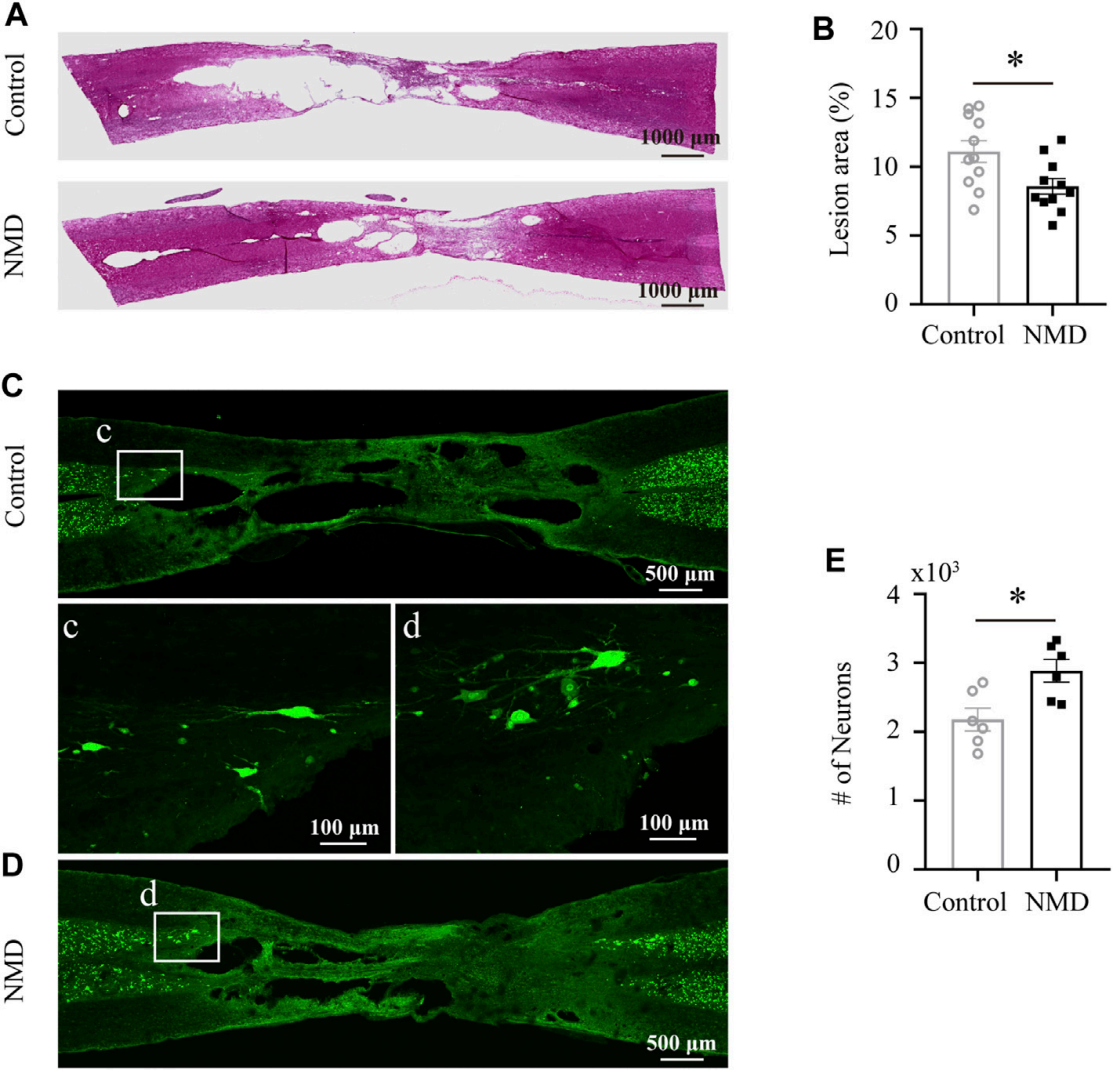
Long-term administration of NMD reduces the lesion area and promotes sparing of perilesional neurons. **(A)** Injured spinal cord stained with HE showing the epicenter of the lesion at 12 weeks postinjury. **(B)** The mean lesion area (%) was quantified by considering the total volume of the spinal cord. NMD-treated rats showed a significantly reduced lesion area relative to controls (*n* = 11 rats in each group). Unpaired t-test, t (20) = 2.628, *p* = 0.0161. **(C–D)** Representative images of horizontal sections of injured spinal cord at 12 weeks post-SCI immunostained with the neuronal marker NeuN. The boxed region in each image is magnified in the corresponding panel c, d to show NeuN + neurons. **(E)** NMD-treated rats showed significantly higher perilesional neuronal sparing than controls (*n* = 6 rats in each group). Unpaired t-test, t (10) = 3.021, *p* = 0.0129.

Several lines of evidence indicate that NMD exerts a neuroprotective effect on different neurological diseases (Singh et al., 2016; Desai et al., 2020; Marcantoni et al., 2020); thus, we investigated the neuroprotective effect of NMD on SCI. We calculated the percent lesion area and found that the percentage in the NMD group (8.57 ± 0.57%, *n* = 11) was significantly lower than that in the control group (11.11 ± 0.78%, *n* = 11) at 12 weeks after SCI [unpaired t-test, t (20) = 2.628, *p* = 0.0161; Figures 4A,B]. Moreover, a significantly greater number of spared perilesional neurons was detected in rats treated with NMD (2883 ± 165.5, *n* = 11) than in the controls (2177 ± 165.1, *n* = 11) [unpaired t-test, t (10) = 3.021, *p* = 0.0129; Figures 4C–E]. These results suggest that long-term treatment with NMD reduces tissue degeneration.

### Long-Term Treatment With Nimodipine Attenuates Gliosis in the Dorsal Horn of the Lumbar Spinal Cord

**FIGURE 5:**
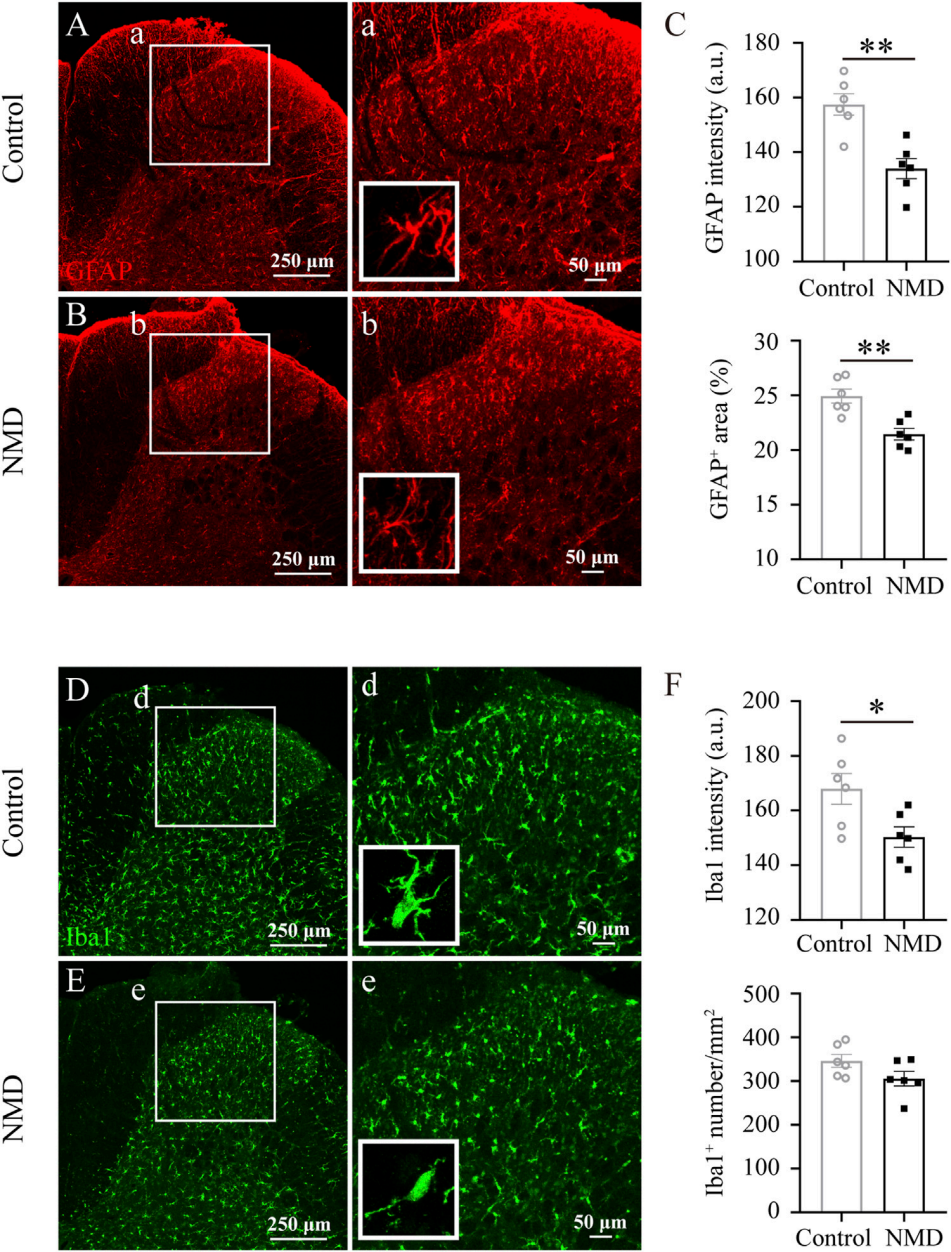
Long-term administration of NMD reduces gliosis in the dorsal horn of the lumbar spinal cord. **(A–B)** Representative images of GFAP immunostaining in the dorsal horn of the lumbar spinal cord in control and NMD-treated rats. The boxed region in each image is magnified in the corresponding panel a, b. NMD treatment attenuated the increases in the area intensity and hypertrophy (insets) compared with controls. **(C)** The GFAP intensity and the percentage of GFAP + area were significantly smaller in the NMD-treated rats than in the controls (*n* = 6 rats in each group). Unpaired t-test, GFAP intensity, t (10) = 4.346, *p* = 0.0015; percentage of GFAP + area, t (10) = 4.147, *p* = 0.002. Arbitrary unit, a.u. **(D–E)** Representative images of Iba1 immunostaining in the dorsal horn of the lumbar spinal cord in control and NMD-treated rats. The boxed region in each image is magnified in the corresponding panel d, e. NMD treatment attenuated the increased area intensity and hypertrophy (insets) compared with controls. **(F)** The Iba1 intensity was significantly reduced in the NMD-treated rats compared with that in the controls (*n* = 6 rats in each group). Unpaired t-test, t (10) = 2.612, *p* = 0.0259. No significant difference in the Iba1+ cell numbers was observed between groups.

In rodent thoracic SCI, astrocytes and microglia are activated chronically in areas remote from the injury, e.g., the dorsal horn of the lumbar spinal cord, and are responsible for the development and maintenance of SCI-induced below-level neuropathic pain (Hains and Waxman, 2006; Hulsebosch et al., 2009; Gwak et al., 2012). In addition, NMD reduces CNS inflammatory responses (Schampel et al., 2017; Zamora et al., 2020). Our behavioral data indicated that long-term treatment with NMD ameliorates pain-related behaviors; thus, we investigated whether NMD reduced astrocyte and microglial activation in the dorsal horn of the lumbar spinal cord. Both astrocytes and microglia exhibited somatic hypertrophy and thickened branches in the control group, indicating an activated phenotype; however, this activated phenotype was less prominent in the NMD group. At the same time, we quantified the intensity of GFAP and found that the intensity in the NMD group (134 ± 3.71, *n* = 6) was significantly lower than that in the control group (157.5 ± 3.92, *n* = 6) [unpaired t-test, t (10) = 4.346, *p* = 0.0015; Figures 5A–C]. Moreover, we also quantified the percentage of GFAP^+^ area and found that the percentage in the NMD group (21.46 ± 0.53%, *n* = 6) was significantly lower than that in the control group (24.94 ± 0.65%, *n* = 6) [unpaired t-test, t (10) = 4.147, *p* = 0.002; Figures 5A–C]. Iba1 staining was performed to detect microglia and showed a significantly lower intensity in the NMD-treated rats (150.3 ± 3.73, *n* = 6) than in the controls (168 ± 5.64, *n* = 6) [unpaired t-test, t (10) = 2.612, *p* = 0.0259; Figures 5D–F]. However, no significant difference in the Iba1^+^ cell numbers was observed between groups [unpaired t-test, t (10) = 1.809, *p* = 0.1; Figures 5D–F]. Thus, long-term treatment with NMD after SCI reduces gliosis in the lumbar spinal cord.

### Long-Term Treatment With Nimodipine Reduces the Calcitonin Gene-Related Peptide Fiber Density in the Lumbar Spinal Cord

**FIGURE 6:**
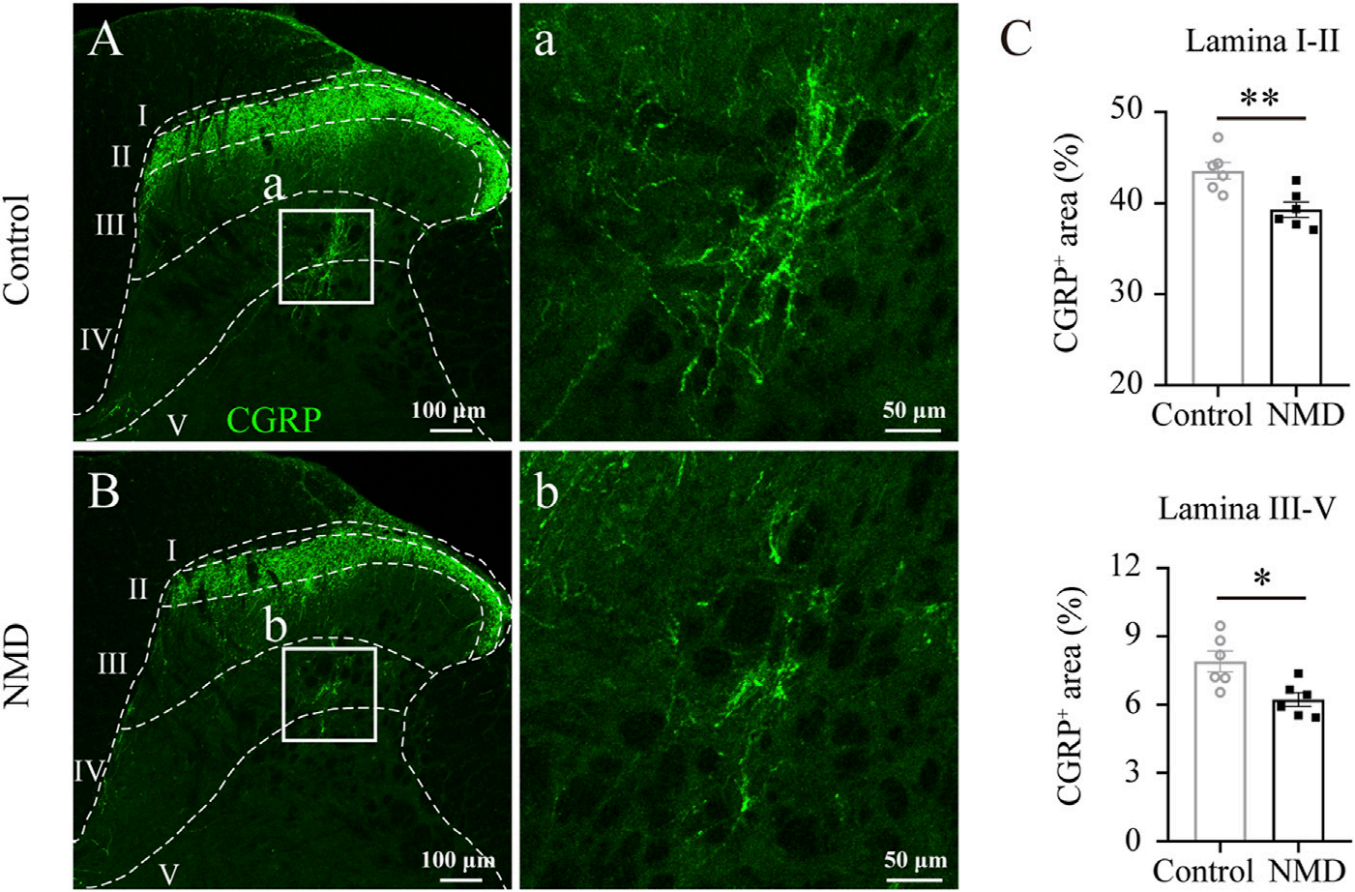
Long-term administration of NMD reduces CGRP^+^ fiber sprouting in the lumbar spinal cord. **(A–B)** Representative images of CGRP immunostaining in the L5 dorsal horn from control or NMD-treated rats. The boxed region in each image is magnified in the corresponding panel a, b showing CGRP^+^ fibers. **(C)** Long-term treatment with NMD reduces the proportional area of CGRP^+^ axons in lamina I–II and lamina III–V (*n* = 6 rats in each group). Unpaired t-test, lamina I–II, t (10) = 3.445, *p* = 0.0063; lamina III–V, t (10) = 3.063, *p* = 0.012.

Previous studies indicate that sprouting of CGRP^+^ sensory afferent fibers into deeper dorsal horn laminae below the lesion site is associated with the development of pain behaviors (Krenz and Weaver, 1998; Ondarza et al., 2003). We quantified CGRP^+^ immunoreactivity profiles in the dorsal horn of the lumbar spinal cord to assess whether NMD reduced the sprouting of CGRP^+^ fibers after SCI. Our analyses revealed a significant reduction in the proportional area of CGRP^+^ axons in laminae I–II [39.3 ± 0.83% versus 43.57 ± 0.92%, t (10) = 3.445, *p* = 0.0063] and laminae III–V [6.23 ± 0.3% versus 7.9 ± 0.46%, t (10) = 3.063, *p* = 0.012] of the NMD group compared with the control group [unpaired t-test, *n* = 6 in each group; Figures 6A–C]. These data indicate that NMD administration after SCI reduces CGRP^+^ axon sprouting in the lumbar spinal cord, which is one of the mechanisms by which NMD therapy attenuates pain-related behaviors.

### Membrane Expression of K^+^–Cl^−^ cotransporter 2 on Lumbar Motor Neurons in NMD-Treated Group is Higher Than in Injured Controls

**FIGURE 7:**
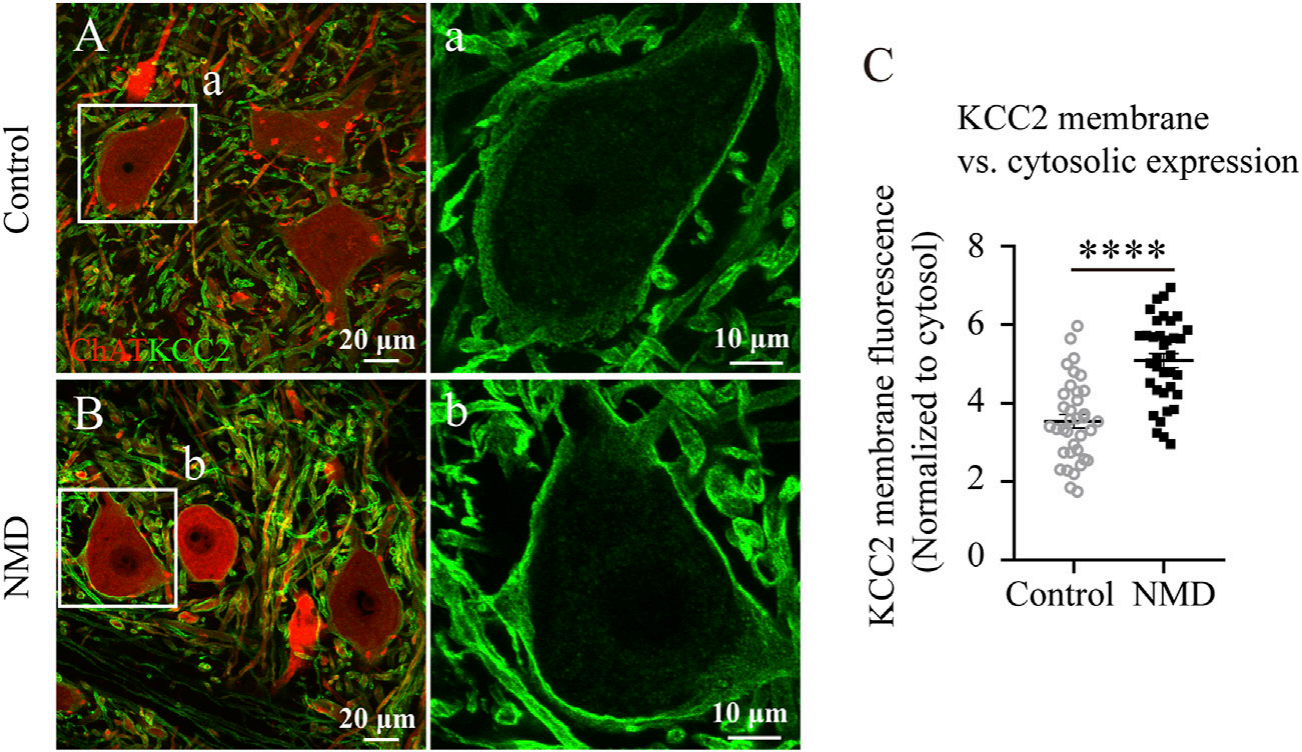
Membrane expression of KCC2 on lumbar motor neurons in NMD-treated group is higher than in injured controls. **(A–B)** Representative images showing ChAT (red) and KCC2 (green) immunolabeling in lumbar motoneurons of control **(A)** or NMD-treated rats **(B)**. The boxed region in each image is magnified in the corresponding panel a, b to show KCC2 immunolabeling. **(C)** Mean pixel intensities of KCC2 motoneuronal membrane immunofluorescence normalized to a determined area of the cytosol. The membrane-cytosol KCC2 ratio was significantly larger in NMD-treated rats than in controls (circles or squares represent individual motoneurons; all groups contained six animals per group, and six motor neurons were analyzed per animal). Unpaired t-test, t (70) = 6.188, *p* < 0.0001.

SCI evokes an increase in intracellular free calcium levels, resulting in calpain activation, which has been suggested to be upstream of the downregulation of KCC2 on motor neurons after SCI (Ray et al., 2003; Plantier et al., 2019). Thus, we reasoned that membrane expression of KCC2 on lumbar motor neurons in NMD-treated group might be higher than in injured controls. We compared the immunofluorescence ratio for membrane and cytosolic expression of KCC2 between the control rats and the NMD-treated rats. Figure 7 shows ChAT and KCC2 immunostaining in lumbar motoneurons at 12 weeks after SCI in control (Figure 7A) or NMD-treated rat (Figure 7B). The membrane-cytosolic KCC2 ratio was significantly larger in the NMD group (5.08 ± 0.18) than in the control group (3.54 ± 0.17) [six motoneurons for each animal, *n* = 6 per group; unpaired t-test, t (70) = 6.188, *p* < 0.0001; Figure 7C]. This result suggests that membrane expression of KCC2 on lumbar motor neurons in NMD treated group is higher than in injured controls.

### Long-Term Treatment With Nimodipine Does Not Mitigate Rat Bladder Dysfunction

**FIGURE 8:**
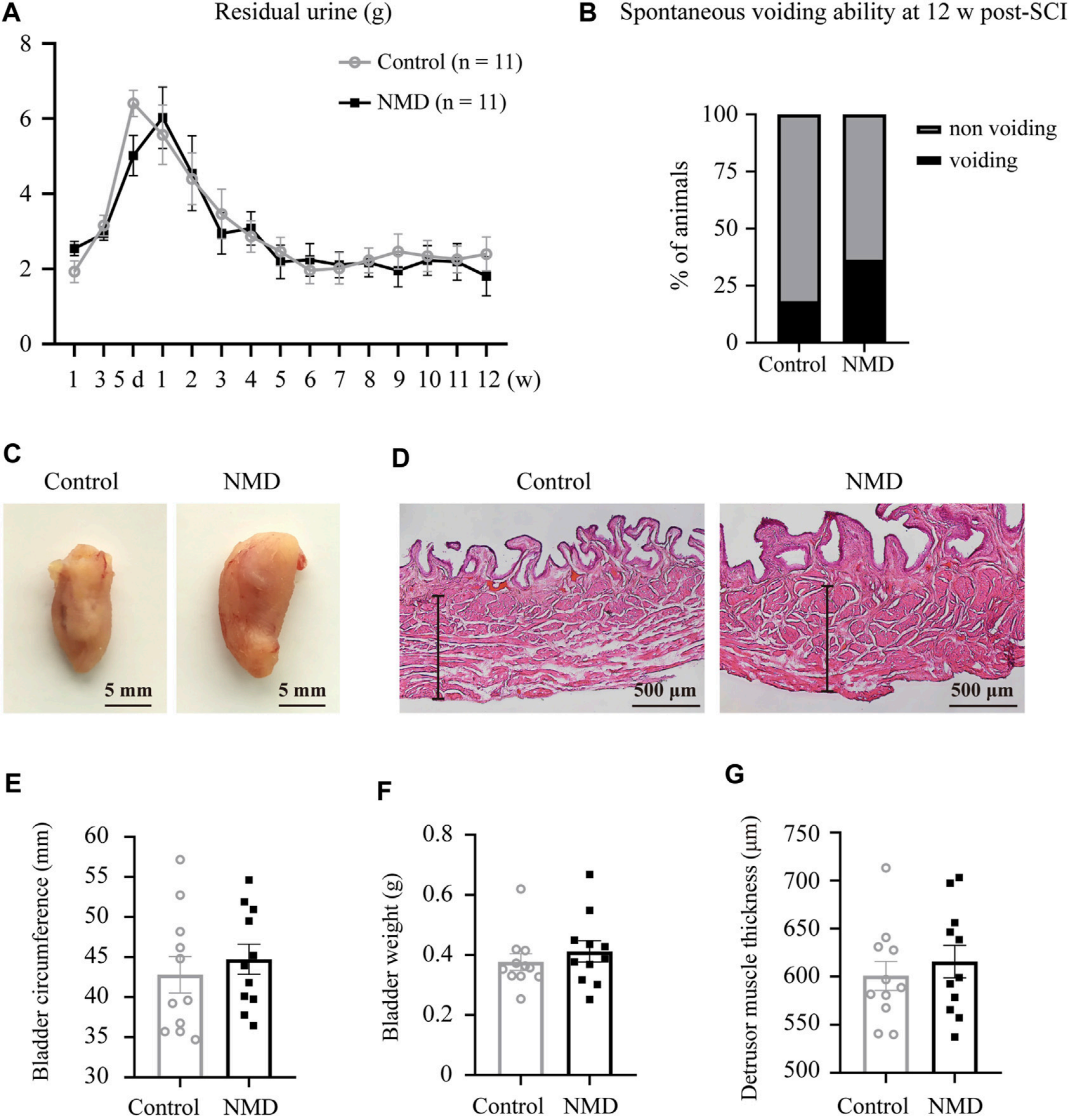
Long-term administration of NMD after SCI does not mitigate rat bladder dysfunction. **(A)** Plot of the residual urine weight over time (*n* = 11 rats in each group). No significant differences were observed between groups using two-way repeated-measures ANOVA [F (1, 20) = 0.1329, *p* > 0.05], but changes over time were significant [F (6.045, 120.9) = 17.12, *p* < 0.0001]. **(B)** At 12 w post-SCI, 36.36% of the NMD treated rats showed spontaneous voiding ability compared to 18.18% of the controls (*n* = 11 rats in each group). Two-tailed Fisher’s exact test, *p* > 0.05. **(C)** Representative images of bladders in control rats or NMD-treated rats. **(D)** Representative images of HE-staining of the bladder wall histology in the two groups. The black lines denote the thickness of the detrusor muscle. **(E–G)** Quantification of the bladder circumference, bladder weight, and thickness of the detrusor muscle in the two groups. Unpaired t-test, *p* > 0.05.

Regaining some level of bladder function is one of the highest priorities for the population with SCI (Anderson, 2004); thus, we also assessed whether long-term treatment with NMD affected bladder dysfunction. We examined spontaneous voiding ability by monitoring each rat daily and weekly to determine whether spontaneous voiding ability had recovered. We also collected and weighed the expressed urine once weekly during routine bladder expression in the morning. No significant differences in the weight of residual urine were observed between groups using two-way repeated-measures ANOVA [F (1, 20) = 0.1329, *p* = 0.7192; Figure 8A]. At 12 weeks post-SCI, 36.36% of the NMD-treated rats showed spontaneous voiding ability compared to 18.18% of the controls (two-tailed Fisher’s exact test, *p* = 0.6351, Figure 8B). Quantification of the bladder circumference, bladder weight, and thickness of the detrusor muscle showed no significant differences between groups (unpaired t-test, *p* > 0.05; Figures 8C–G). These data indicate that long-term treatment with NMD after SCI does not mitigate rat bladder dysfunction.

## Discussion

As shown in the present study, long-term administration of NMD after SCI improved locomotion, pain-related behaviors and spasticity-like symptoms in rats. We further illustrated that this pharmacological treatment resulted in higher tissue preservation, perilesional neuronal sparing, and membrane expression of KCC2 on lumbar motor neurons, and reduced gliosis and CGRP^+^ fiber sprouting in the lumbar spinal cord. These results suggest that NMD may be a potential therapeutic option for the treatment of SCI.

NMD efficiently passes the blood-brain barrier (Langley and Sorkin, 1989; Tomassoni et al., 2008). Thus, it has been increasingly recognized as a potential therapeutic approach for neurological diseases, including stroke, neurodegenerative diseases, traumatic brain injury, and SCI (Carlson et al., 2020; Desai et al., 2020; Marcantoni et al., 2020). NMD mainly acts on L-/T-type calcium channels (Gurkoff et al., 2013). In the CNS, subtypes of these channels, such as Cav1.2 or Cav1.3, are mainly expressed on neurons, where they shape neuronal firing and activate Ca^2+^-dependent pathways involved in the control of gene expression (Zamponi et al., 2015). Although the evidence is not strong, a previous study suggested that acute treatment with NMD for 1 week may improve neurological recovery after SCI (Ross et al., 1993). However, the T1 segment was compressed in that study, and the administration of NMD may worsen cardiovascular dysfunction, such as unstable blood pressure and hypotension, that develop after high-level SCI (Phillips and Krassioukov, 2015). Additionally, a study using equivalent doses for humans in baboons has shown that the administration of NMD for 1 week after SCI enhances spinal cord blood flow and limits the size of the spinal cord lesion (Pointillart et al., 1993). Consistent with previous reports, we found a significantly smaller lesion size, greater perilesional neuronal sparing and better locomotor function in rats treated with NMD than in the control rats. Increased tissue sparing is associated with higher locomotor scores (Basso et al., 1996). Thus, the effect of NMD on locomotor scores may be explained by increased tissue sparing. We observed that the effect of NMD on BBB scores was evident during the chronic phase of recovery. In fact, 25 mm contusion injury is a moderate-to-severe SCI model because the rats display flaccid paralysis early after injury and some rats do not regain the ability to take weight supported steps at 6 weeks after SCI (Basso et al., 1996). Although NMD may have some effect, however, the damage is severe early after 25 mm SCI which may outweigh the therapeutic effect of NMD. Unlike another calcium channel blocker GBP (Sun et al., 2020), NMD improves locomotor function without affecting spontaneous open-field activity. We chose to administer NMD for 6 weeks because a previous study suggested that 6 weeks treatment may blunt secondary injury changes (Marcantoni et al., 2020). However, further studies are required to explore the safety and efficacy of longer treatment duration such as 12 weeks treatment. Of note is that the locomotor recovery for control animals in the present study is somewhat worse than that of untreated animals subjected to similar severity of injury (25 mm contusion) in previous study (Basso et al., 1996). Strain and BW differences may account for different outcomes between the current and previous work. Notably, rats recover from SCI in a strain-specific manner (Mills et al., 2001; Kjell and Olson, 2016). In addition, the overall size of the thoracic spinal cord changes with the size of rats and the same contusion on a larger spinal cord produces a milder damage than on a smaller spinal cord (Fouad et al., 2020). In the present study Sprague-Dawley rats weighing 260–300 g were used, whereas in the previous study Long-Evans rats weighing 265–415 g were used. We recorded hind limb grip strength and open-field spontaneous activity after SCI to assess the possible side effect of NMD, but no differences were observed in these measurements between groups. Together, these results indicate that long-term therapy with NMD exerts neuroprotective effects after SCI.

The application of the Von Frey test requires weight support, as evidenced by a score of 9 during BBB locomotor scoring (Detloff et al., 2010; Fouad et al., 2020). This criterion ensures that the behavioral test match but not surpass the functional capacity of the animals being tested (Fouad et al., 2020). In the present study, most SCI-rats in NMD (10/11) and control groups (9/11) recovered to this level (BBB score 9) at week 6 after injury. Therefore, sensory tests began at week 6 after SCI. The effect of NMD on neuropathic pain seems to be explained by multiple factors. First, strong evidence that L-/T-type calcium channels are involved in neuropathic pain has been documented (Favereaux et al., 2011; Garcia-Caballero et al., 2014; Radwani et al., 2016). Thus, the blockade of these calcium channels may exert analgesic effects. Second, several reports have shown that NMD may suppress astrocyte and microglial activation (Schampel et al., 2017; Zamora et al., 2020). Indeed, a previous study suggested that the administration of NMD for 7 days attenuates spinal cord inflammation after SCI (Jia et al., 2015). Based on our data, the intensity of GFAP and Iba1 staining and the percentage of GFAP^+^ area in the dorsal horn of the lumbar cord were significantly decreased in animals treated with NMD compared to the controls at 12 weeks after SCI. Thus, the regulation of astrocytes or microglial function may also contribute to the restored nociceptive thresholds observed in the NMD group. Third, sprouting of CGRP^+^ fibers into deeper dorsal horn laminae below the lesion site is associated with the development of pain behaviors (Krenz and Weaver, 1998; Ondarza et al., 2003). We observed that the sprouting of CGRP^+^ fibers in the lumbar spinal cord was reduced in NMD-treated rats compared with controls. This phenomenon is similar to previous study using GBP to treat maladaptive plasticity after SCI (Brennan et al., 2021), however, the detailed mechanisms that both drugs inhibit CGRP^+^ sensory fiber growth remain to be investigated. We noted that the Von Frey test was not significant different between the two groups at week 8 but was prior and afterwards. The possible reason is that the variations of the pain-related behaviors at some time point are too large (Kjell et al., 2013; Dugan and Sagen, 2015; Gaudet et al., 2017). Together, these results suggest that long-term treatment with NMD after SCI attenuates pain-related behaviors.

One of the novel findings in this study was that the membrane expression of KCC2 on lumbar motor neurons in NMD treated group is higher than in injured controls. Following SCI, the increased intracellular calcium concentration and excessive calcium influx through L-type calcium channels may promote the activation of calcium-dependent calpains to decrease KCC2 expression on the lumbar motoneuronal membrane (Boulenguez et al., 2010; Plantier et al., 2019). Thus, the administration of NMD may mitigate this phenomenon by blocking calcium channels. Indeed, a significantly higher membrane-cytosol ratio of KCC2 expression on the lumbar motor neurons was observed in the NMD-treated rats than in the controls at 12 weeks after SCI. A reduction in KCC2 expression on the lumbar motor neurons after SCI is associated with spasticity (Boulenguez et al., 2010; Plantier et al., 2019). In our study, NMD-treated rats showed attenuated spasticity-like symptoms, as evidenced by the restored RDD of the H-reflex. Consistent with a previous report (Marcantoni et al., 2020), these data suggest that long-term treatment with NMD after SCI might be a potential therapeutic strategy for the treatment of spasticity.

Long-term administration of NMD did not result in significant difference in survival rate, BW, hind limb grip strength, and the open-field spontaneous activity between the two groups, suggesting that NMD may be used in a long term. However, the safety and the possible side effects of the treatment needs further investigation. In addition, NMD treatment did not mitigate bladder dysfunction after SCI. Perhaps because the calcium channels expressed on bladder muscle are associated with muscle contraction (Zamponi et al., 2015), blockade of these channels may induce partial bladder weakness; however, this hypothesis remains to be thoroughly examined.

Our study has several limitations. First, we only used female rats because manual expression of the bladder is more easily accomplished due to the shorter and wider urethra in females than in male rats. Thus, further exploration is required to determine whether long-term therapy with NMD promotes functional recovery in male rats. In addition, it remains unknown whether estrous cycle adds to variability in pain-related behaviors (Mogil and Chanda, 2005). Therefore, estrous cycle need to be monitored in the future study to ascertain whether the stage of estrus could have any implications in the treatment efficiency or possible variations. Second, as noted above, high-level SCI, especially injuries at or above the T6 spinal segments, may result in neurogenic shock, e.g., hypotension. Therefore, the use of NMD alone to treat high-level SCI seems to be limited. Third, we did not examine the route and the optimal dose of NMD for SCI. However, after considering allometric scaling, the dose of 10 mg/kg BW in rats would be equivalent to 1.6 mg/kg BW in humans (Nair and Jacob, 2016), which is in the normal range of approved doses of NMD for patients with SAH (Steiner et al., 2013). Thus, the dose used in this study seems reasonable. In addition, oral intake of NMD should also be investigated, since NMD administration through this route has been approved.

## Conclusion

This study suggests that long-term treatment with NMD can promote functional recovery after SCI in rats, presumably due to its actions on tissue preservation, gliosis and CGRP^+^ fiber sprouting in the lumbar spinal cord, and expression of KCC2 on lumbar motor neurons. Due to the increasing interest in its possible usefulness in neurological diseases (Schampel et al., 2017; Carlson et al., 2020; Desai et al., 2020; Marcantoni et al., 2020), our data indicate that NMD may merit additional study as a possible therapeutic agent for the treatment of SCI.

## Data Availability

The original contributions presented in the study are included in the article/Supplementary Material, further inquiries can be directed to the corresponding author.

## Supplementary Material

Supplementary Figure 1 Progression of weight gain over 12 weeks after SCI in NMD-treated group and control group.

## Abbreviations

SCI, spinal cord injury; NMD, nimodipine; SAH, subarachnoid haemorrhage; CNS, central nervous system; BBB score, Basso, Beattie, and Bresnahan open-field locomotor score; H-reflex, Hoffmann reflex; RDD, rate-dependent depression; BW, body weight; PFA, paraformaldehyde; HE, hematoxylin and eosin; ROI, region of interest; CGRP, calcitonin gene-related peptide; KCC2, K+–Cl− cotransporter 2; ChAT, choline acetyltransferase; GBP, gabapentin.

## References

[B1] Ahuja C. S. Martin A. R. Fehlings M. (2016). Recent Advances in Managing a Spinal Cord Injury Secondary to Trauma. F1000Res 5, F1000Research. 10.12688/f1000research.7586.1 PMC4890313 27303644

[B2] Ahuja C. S. Wilson J. R. Nori S. Kotter M. R. N. Druschel C. Curt A. (2017). Traumatic Spinal Cord Injury. Nat. Rev. Dis. Primers 3, 17018. 10.1038/nrdp.2017.18 28447605

[B3] Anderson K. D. (2004). Targeting Recovery: Priorities of the Spinal Cord-Injured Population. J. Neurotrauma 21, 1371–1383. 10.1089/neu.2004.21.1371 15672628

[B4] Andresen S. R. Biering-Sørensen F. Hagen E. M. Nielsen J. F. Bach F. W. Finnerup N. B. (2016). Pain, Spasticity and Quality of Life in Individuals with Traumatic Spinal Cord Injury in Denmark. Spinal Cord 54, 973–979. 10.1038/sc.2016.46 27067654

[B5] Basso D. M. Beattie M. S. Bresnahan J. C. (1995). A Sensitive and Reliable Locomotor Rating Scale for Open Field Testing in Rats. J. Neurotrauma 12, 1–21. 10.1089/neu.1995.12.1 7783230

[B6] Basso D. M. Beattie M. S. Bresnahan J. C. (1996). Graded Histological and Locomotor Outcomes after Spinal Cord Contusion Using the NYU Weight-Drop Device versus Transection. Exp. Neurol. 139, 244–256. 10.1006/exnr.1996.0098 8654527

[B7] Bilchak J. N. Yeakle K. Caron G. Malloy D. Côté M. P. (2021). Enhancing KCC2 Activity Decreases Hyperreflexia and Spasticity after Chronic Spinal Cord Injury. Exp. Neurol. 338, 113605. 10.1016/j.expneurol.2021.113605 33453210 PMC7904648

[B8] Boulenguez P. Liabeuf S. Bos R. Bras H. Jean-Xavier C. Brocard C. (2010). Down-regulation of the Potassium-Chloride Cotransporter KCC2 Contributes to Spasticity after Spinal Cord Injury. Nat. Med. 16, 302–307. 10.1038/nm.2107 20190766

[B9] Brennan F. H. Noble B. T. Wang Y. Guan Z. Davis H. Mo X. (2021). Acute post-injury Blockade of α2δ-1 Calcium Channel Subunits Prevents Pathological Autonomic Plasticity after Spinal Cord Injury. Cell Rep 34, 108667. 10.1016/j.celrep.2020.108667 33503436 PMC8817229

[B10] Carlson A. P. Hänggi D. Macdonald R. L. Shuttleworth C. W. (2020). Nimodipine Reappraised: An Old Drug with a Future. Curr. Neuropharmacol. 18, 65–82. 10.2174/1570159X17666190927113021 31560289 PMC7327937

[B11] Desai R. A. Davies A. L. Del Rossi N. Tachrount M. Dyson A. Gustavson B. (2020). Nimodipine Reduces Dysfunction and Demyelination in Models of Multiple Sclerosis. Ann. Neurol. 88, 123–136. 10.1002/ana.25749 32293054 PMC7737229

[B12] Detloff M. R. Clark L. M. Hutchinson K. J. Kloos A. D. Fisher L. C. Basso D. M. (2010). Validity of Acute and Chronic Tactile Sensory Testing after Spinal Cord Injury in Rats. Exp. Neurol. 225, 366–376. 10.1016/j.expneurol.2010.07.009 20643128 PMC4933012

[B13] Dugan E. A. Sagen J. (2015). An Intensive Locomotor Training Paradigm Improves Neuropathic Pain Following Spinal Cord Compression Injury in Rats. J. Neurotrauma 32, 622–632. 10.1089/neu.2014.3692 25539034

[B14] Fan C. Zheng Y. Cheng X. Qi X. Bu P. Luo X. (2013). Transplantation of D15A-Expressing Glial-Restricted-Precursor-Derived Astrocytes Improves Anatomical and Locomotor Recovery after Spinal Cord Injury. Int. J. Biol. Sci. 9, 78–93. 10.7150/ijbs.5626 23289019 PMC3535536

[B15] Fandel T. M. Trivedi A. Nicholas C. R. Zhang H. Chen J. Martinez A. F. (2016). Transplanted Human Stem Cell-Derived Interneuron Precursors Mitigate Mouse Bladder Dysfunction and Central Neuropathic Pain after Spinal Cord Injury. Cell stem cell 19, 544–557. 10.1016/j.stem.2016.08.020 27666009

[B16] Favereaux A. Thoumine O. Bouali-Benazzouz R. Roques V. Papon M. A. Salam S. A. (2011). Bidirectional Integrative Regulation of Cav1.2 Calcium Channel by microRNA miR-103: Role in Pain. EMBO J. 30, 3830–3841. 10.1038/emboj.2011.249 21804529 PMC3173784

[B17] Finnerup N. B. Norrbrink C. Trok K. Piehl F. Johannesen I. L. Sørensen J. C. (2014). Phenotypes and Predictors of Pain Following Traumatic Spinal Cord Injury: a Prospective Study. J. Pain 15, 40–48. 10.1016/j.jpain.2013.09.008 24268112

[B18] Finnerup N. B. (2013). Pain in Patients with Spinal Cord Injury. Pain 154 (Suppl. 1), S71–S76. 10.1016/j.pain.2012.12.007 23375163

[B19] Fouad K. Ng C. Basso D. M. (2020). Behavioral Testing in Animal Models of Spinal Cord Injury. Exp. Neurol. 333, 113410. 10.1016/j.expneurol.2020.113410 32735871 PMC8325780

[B20] García-Caballero A. Gadotti V. M. Stemkowski P. Weiss N. Souza I. A. Hodgkinson V. (2014). The Deubiquitinating Enzyme USP5 Modulates Neuropathic and Inflammatory Pain by Enhancing Cav3.2 Channel Activity. Neuron 83, 1144–1158. 10.1016/j.neuron.2014.07.036 25189210

[B21] Gaudet A. D. Ayala M. T. Schleicher W. E. Smith E. J. Bateman E. M. Maier S. F. (2017). Exploring Acute-To-Chronic Neuropathic Pain in Rats after Contusion Spinal Cord Injury. Exp. Neurol. 295, 46–54. 10.1016/j.expneurol.2017.05.011 28552717

[B22] Gong C. Zheng X. Guo F. Wang Y. Zhang S. Chen J. (2021). Human Spinal GABA Neurons Alleviate Spasticity and Improve Locomotion in Rats with Spinal Cord Injury. Cel Rep 34, 108889. 10.1016/j.celrep.2021.108889 33761348

[B23] Grey M. J. Klinge K. Crone C. Lorentzen J. Biering-Sørensen F. Ravnborg M. (2008). Post-activation Depression of Soleus Stretch Reflexes in Healthy and Spastic Humans. Exp. Brain Res. 185, 189–197. 10.1007/s00221-007-1142-6 17932663

[B24] Gurkoff G. Shahlaie K. Lyeth B. Berman R. (2013). Voltage-gated Calcium Channel Antagonists and Traumatic Brain Injury. Pharmaceuticals (Basel) 6, 788–812. 10.3390/ph6070788 24276315 PMC3816709

[B25] Gwak Y. S. Kang J. Unabia G. C. Hulsebosch C. E. (2012). Spatial and Temporal Activation of Spinal Glial Cells: Role of Gliopathy in central Neuropathic Pain Following Spinal Cord Injury in Rats. Exp. Neurol. 234, 362–372. 10.1016/j.expneurol.2011.10.010 22036747 PMC3303938

[B26] Hains B. C. Waxman S. G. (2006). Activated Microglia Contribute to the Maintenance of Chronic Pain after Spinal Cord Injury. J. Neurosci. 26, 4308–4317. 10.1523/JNEUROSCI.0003-06.2006 16624951 PMC6674010

[B27] Huang G. Lee X. Bian Y. Shao Z. Sheng G. Pepinsky R. B. (2013). Death Receptor 6 (DR6) Antagonist Antibody Is Neuroprotective in the Mouse SOD1G93A Model of Amyotrophic Lateral Sclerosis. Cell Death Dis 4, e841. 10.1038/cddis.2013.378 24113175 PMC3824687

[B28] Hulsebosch C. E. Hains B. C. Crown E. D. Carlton S. M. (2009). Mechanisms of Chronic central Neuropathic Pain after Spinal Cord Injury. Brain Res. Rev. 60, 202–213. 10.1016/j.brainresrev.2008.12.010 19154757 PMC2796975

[B29] Hultborn H. Illert M. Nielsen J. Paul A. Ballegaard M. Wiese H. (1996). On the Mechanism of the post-activation Depression of the H-Reflex in Human Subjects. Exp. Brain Res. 108, 450–462. 10.1007/BF00227268 8801125

[B30] Jia Y. F. Gao H. L. Ma L. J. Li J. (2015). Effect of Nimodipine on Rat Spinal Cord Injury. Genet. Mol. Res. 14, 1269–1276. 10.4238/2015.February.13.5 25730065

[B31] Kjell J. Olson L. (2016). Rat Models of Spinal Cord Injury: from Pathology to Potential Therapies. Dis. Model. Mech. 9, 1125–1137. 10.1242/dmm.025833 27736748 PMC5087825

[B32] Kjell J. Sandor K. Josephson A. Svensson C. I. Abrams M. B. (2013). Rat Substrains Differ in the Magnitude of Spontaneous Locomotor Recovery and in the Development of Mechanical Hypersensitivity after Experimental Spinal Cord Injury. J. Neurotrauma 30, 1805–1811. 10.1089/neu.2013.2998 23879467 PMC3804226

[B33] Kramer S. M. May J. R. Patrick D. J. Chouinard L. Boyer M. Doyle N. (2009). Instilled or Injected Purified Natural Capsaicin Has No Adverse Effects on Rat Hindlimb Sensory-Motor Behavior or Osteotomy Repair. Anesth. Analg. 109, 249–257. 10.1213/ane.0b013e3181a7f589 19535718

[B34] Krause J. S. Carter R. E. Pickelsimer E. (2009). Behavioral Risk Factors of Mortality after Spinal Cord Injury. Arch. Phys. Med. Rehabil. 90, 95–101. 10.1016/j.apmr.2008.07.012 19154835 PMC2813690

[B35] Krenz N. R. Weaver L. C. (1998). Sprouting of Primary Afferent Fibers after Spinal Cord Transection in the Rat. Neuroscience 85, 443–458. 10.1016/s0306-4522(97)00622-2 9622243

[B36] Langley M. S. Sorkin E. M. (1989). Nimodipine. A Review of its Pharmacodynamic and Pharmacokinetic Properties, and Therapeutic Potential in Cerebrovascular Disease. Drugs 37, 669–699. 10.2165/00003495-198937050-00004 2663415

[B37] Li X. Yu Z. Zong W. Chen P. Li J. Wang M. (2020). Deficiency of the Microglial Hv1 Proton Channel Attenuates Neuronal Pyroptosis and Inhibits Inflammatory Reaction after Spinal Cord Injury. J. Neuroinflammation 17, 263. 10.1186/s12974-020-01942-x 32891159 PMC7487532

[B38] Liabeuf S. Stuhl-Gourmand L. Gackière F. Mancuso R. Sanchez Brualla I. Marino P. (2017). Prochlorperazine Increases KCC2 Function and Reduces Spasticity after Spinal Cord Injury. J. Neurotrauma 34, 3397–3406. 10.1089/neu.2017.5152 28747093

[B39] Macdonald R. L. Schweizer T. A. (2017). Spontaneous Subarachnoid Haemorrhage. Lancet 389, 655–666. 10.1016/S0140-6736(16)30668-7 27637674

[B40] Marcantoni M. Fuchs A. Löw P. Bartsch D. Kiehn O. Bellardita C. (2020). Early Delivery and Prolonged Treatment with Nimodipine Prevents the Development of Spasticity after Spinal Cord Injury in Mice. Sci. Transl. Med. 12. 10.1126/scitranslmed.aay0167 32295897

[B41] Mills C. D. Hains B. C. Johnson K. M. Hulsebosch C. E. (2001). Strain and Model Differences in Behavioral Outcomes after Spinal Cord Injury in Rat. J. Neurotrauma 18, 743–756. 10.1089/089771501316919111 11526981

[B42] Mogil J. S. Chanda M. L. (2005). The Case for the Inclusion of Female Subjects in Basic Science Studies of Pain. Pain 117, 1–5. 10.1016/j.pain.2005.06.020 16098670

[B43] Mothe A. J. Coelho M. Huang L. Monnier P. P. Cui Y. F. Mueller B. K. (2020). Delayed Administration of the Human Anti-RGMa Monoclonal Antibody Elezanumab Promotes Functional Recovery Including Spontaneous Voiding after Spinal Cord Injury in Rats. Neurobiol. Dis. 143, 104995. 10.1016/j.nbd.2020.104995 32590037

[B44] Nair A. B. Jacob S. (2016). A Simple Practice Guide for Dose Conversion between Animals and Human. J. Basic Clin. Pharm. 7, 27–31. 10.4103/0976-0105.177703 27057123 PMC4804402

[B45] Ondarza A. B. Ye Z. Hulsebosch C. E. (2003). Direct Evidence of Primary Afferent Sprouting in Distant Segments Following Spinal Cord Injury in the Rat: Colocalization of GAP-43 and CGRP. Exp. Neurol. 184, 373–380. 10.1016/j.expneurol.2003.07.002 14637107

[B46] Parada C. A. Vivancos G. G. Tambeli C. H. Cunha F. Q. Ferreira S. H. (2003). Activation of Presynaptic NMDA Receptors Coupled to NaV1.8-resistant Sodium Channel C-Fibers Causes Retrograde Mechanical Nociceptor Sensitization. Proc. Natl. Acad. Sci. U. S. A. 100, 2923–2928. 10.1073/pnas.252777799 12589028 PMC151442

[B47] Phillips A. A. Krassioukov A. V. (2015). Contemporary Cardiovascular Concerns after Spinal Cord Injury: Mechanisms, Maladaptations, and Management. J. Neurotrauma 32, 1927–1942. 10.1089/neu.2015.3903 25962761

[B48] Plantier V. Sanchez-Brualla I. Dingu N. Brocard C. Liabeuf S. Gackière F. (2019). Calpain Fosters the Hyperexcitability of Motoneurons after Spinal Cord Injury and Leads to Spasticity. eLife 8. 10.7554/eLife.51404 PMC6927741 31815668

[B49] Pointillart V. Gense D. Gross C. Bidabé A. M. Gin A. M. Rivel J. (1993). Effects of Nimodipine on Posttraumatic Spinal Cord Ischemia in Baboons. J. Neurotrauma 10, 201–213. 10.1089/neu.1993.10.201 8411220

[B50] Pointillart V. Petitjean M. E. Wiart L. Vital J. M. Lassié P. Thicoipé M. (2000). Pharmacological Therapy of Spinal Cord Injury during the Acute Phase. Spinal Cord 38, 71–76. 10.1038/sj.sc.3100962 10762178

[B51] Radwani H. Lopez-Gonzalez M. J. Cattaert D. Roca-Lapirot O. Dobremez E. Bouali-Benazzouz R. (2016). Cav1.2 and Cav1.3 L-type Calcium Channels Independently Control Short- and Long-Term Sensitization to Pain. J. Physiol. 594, 6607–6626. 10.1113/JP272725 27231046 PMC5108908

[B52] Ray S. K. Hogan E. L. Banik N. L. (2003). Calpain in the Pathophysiology of Spinal Cord Injury: Neuroprotection with Calpain Inhibitors. Brain Res. Brain Res. Rev. 42, 169–185. 10.1016/s0165-0173(03)00152-8 12738057

[B53] Ross I. B. Tator C. H. Theriault E. (1993). Effect of Nimodipine or Methylprednisolone on Recovery from Acute Experimental Spinal Cord Injury in Rats. Surg. Neurol. 40, 461–470. 10.1016/0090-3019(93)90048-6 8235968

[B54] Ryu Y. Ogata T. Nagao M. Sawada Y. Nishimura R. Fujita N. (2018). Effects of Treadmill Training Combined with Serotonergic Interventions on Spasticity after Contusive Spinal Cord Injury. J. Neurotrauma 35, 1358–1366. 10.1089/neu.2017.5400 29336209

[B55] Sandner B. Puttagunta R. Motsch M. Bradke F. Ruschel J. Blesch A. (2018). Systemic Epothilone D Improves Hindlimb Function after Spinal Cord Contusion Injury in Rats. Exp. Neurol. 306, 250–259. 10.1016/j.expneurol.2018.01.018 29408734

[B56] Schampel A. Volovitch O. Koeniger T. Scholz C. J. Jörg S. Linker R. A. (2017). Nimodipine Fosters Remyelination in a Mouse Model of Multiple Sclerosis and Induces Microglia-specific Apoptosis. Proc. Natl. Acad. Sci. U. S. A. 114, E3295–E3304. 10.1073/pnas.1620052114 28381594 PMC5402421

[B57] Sharp K. G. Yee K. M. Steward O. (2014). A Re-assessment of Long Distance Growth and Connectivity of Neural Stem Cells after Severe Spinal Cord Injury. Exp. Neurol. 257, 186–204. 10.1016/j.expneurol.2014.04.008 24747827 PMC4123968

[B58] Silva N. A. Sousa N. Reis R. L. Salgado A. J. (2014). From Basics to Clinical: a Comprehensive Review on Spinal Cord Injury. Prog. Neurobiol. 114, 25–57. 10.1016/j.pneurobio.2013.11.002 24269804

[B59] Singh A. Verma P. Balaji G. Samantaray S. Mohanakumar K. P. (2016). Nimodipine, an L-type Calcium Channel Blocker Attenuates Mitochondrial Dysfunctions to Protect against 1-Methyl-4-Phenyl-1,2,3,6-Tetrahydropyridine-Induced Parkinsonism in Mice. Neurochem. Int. 99, 221–232. 10.1016/j.neuint.2016.07.003 27395789

[B60] Steiner T. Juvela S. Unterberg A. Jung C. Forsting M. Rinkel G. (2013). European Stroke Organization Guidelines for the Management of Intracranial Aneurysms and Subarachnoid Haemorrhage. Cerebrovasc. Dis. 35, 93–112. 10.1159/000346087 23406828

[B61] Sun W. Larson M. J. Kiyoshi C. M. Annett A. J. Stalker W. A. Peng J. (2020). Gabapentinoid Treatment Promotes Corticospinal Plasticity and Regeneration Following Murine Spinal Cord Injury. J. Clin. Invest. 130, 345–358. 10.1172/JCI130391 31793909 PMC6934190

[B62] Tator C. H. Fehlings M. G. (1991). Review of the Secondary Injury Theory of Acute Spinal Cord Trauma with Emphasis on Vascular Mechanisms. J. Neurosurg. 75, 15–26. 10.3171/jns.1991.75.1.0015 2045903

[B63] Tator C. H. Hashimoto R. Raich A. Norvell D. Fehlings M. G. Harrop J. S. (2012). Translational Potential of Preclinical Trials of Neuroprotection through Pharmacotherapy for Spinal Cord Injury. J. Neurosurg. Spine 17, 157–229. 10.3171/2012.5.AOSPINE12116 22985382

[B64] Thompson F. J. Reier P. J. Lucas C. C. Parmer R. (1992). Altered Patterns of Reflex Excitability Subsequent to Contusion Injury of the Rat Spinal Cord. J. Neurophysiol. 68, 1473–1486. 10.1152/jn.1992.68.5.1473 1479425

[B65] Tomassoni D. Lanari A. Silvestrelli G. Traini E. Amenta F. (2008). Nimodipine and its Use in Cerebrovascular Disease: Evidence from Recent Preclinical and Controlled Clinical Studies. Clin. Exp. Hypertens. 30, 744–766. 10.1080/10641960802580232 19021025

[B66] Walker C. L. Fry C. M. E. Wang J. Du X. Zuzzio K. Liu N. K. (2019). Functional and Histological Gender Comparison of Age-Matched Rats after Moderate Thoracic Contusive Spinal Cord Injury. J. Neurotrauma 36, 1974–1984. 10.1089/neu.2018.6233 30489213 PMC6599384

[B67] Watson C. Paxinos G. Kayalioglu G. Heise C. (2009). Atlas of the Rat Spinal Cord-Chapter 15" in the Spinal Cord A Christopher and Dana Reeve Foundation Text and Atlas, 238–306. 10.1016/b978-0-12-374247-6.50019-5

[B68] Yamazaki K. Kawabori M. Seki T. Takamiya S. Tateno T. Konno K. (2020). FTY720 Attenuates Neuropathic Pain after Spinal Cord Injury by Decreasing Systemic and Local Inflammation in a Rat Spinal Cord Compression Model. J. Neurotrauma 37, 1720–1728. 10.1089/neu.2019.6905 32216535 PMC7368387

[B69] Zamora N. N. Cheli V. T. Santiago González D. A. Wan R. Paez P. M. (2020). Deletion of Voltage-Gated Calcium Channels in Astrocytes during Demyelination Reduces Brain Inflammation and Promotes Myelin Regeneration in Mice. J. Neurosci. 40, 3332–3347. 10.1523/JNEUROSCI.1644-19.2020 32169969 PMC7178909

[B70] Zamponi G. W. Striessnig J. Koschak A. Dolphin A. C. (2015). The Physiology, Pathology, and Pharmacology of Voltage-Gated Calcium Channels and Their Future Therapeutic Potential. Pharmacol. Rev. 67, 821–870. 10.1124/pr.114.009654 26362469 PMC4630564

[B71] Zuo C. Cao H. Ding F. Zhao J. Huang Y. Li G. (2020). Neuroprotective Efficacy of Different Levels of High-Frequency Repetitive Transcranial Magnetic Stimulation in Mice with CUMS-Induced Depression: Involvement of the p11/BDNF/Homer1a Signaling Pathway. J. Psychiatr. Res. 125, 152–163. 10.1016/j.jpsychires.2020.03.018 32289652

