# Supplementary figures and images for: Nimodipine Promotes Functional Recovery After Spinal Cord Injury in Rats
Source: Front Pharmacol. 2021 Sep 13;12:733420. doi: 10.3389/fphar.2021.733420 (PMC8477750; doi:10.3389/fphar.2021.733420)

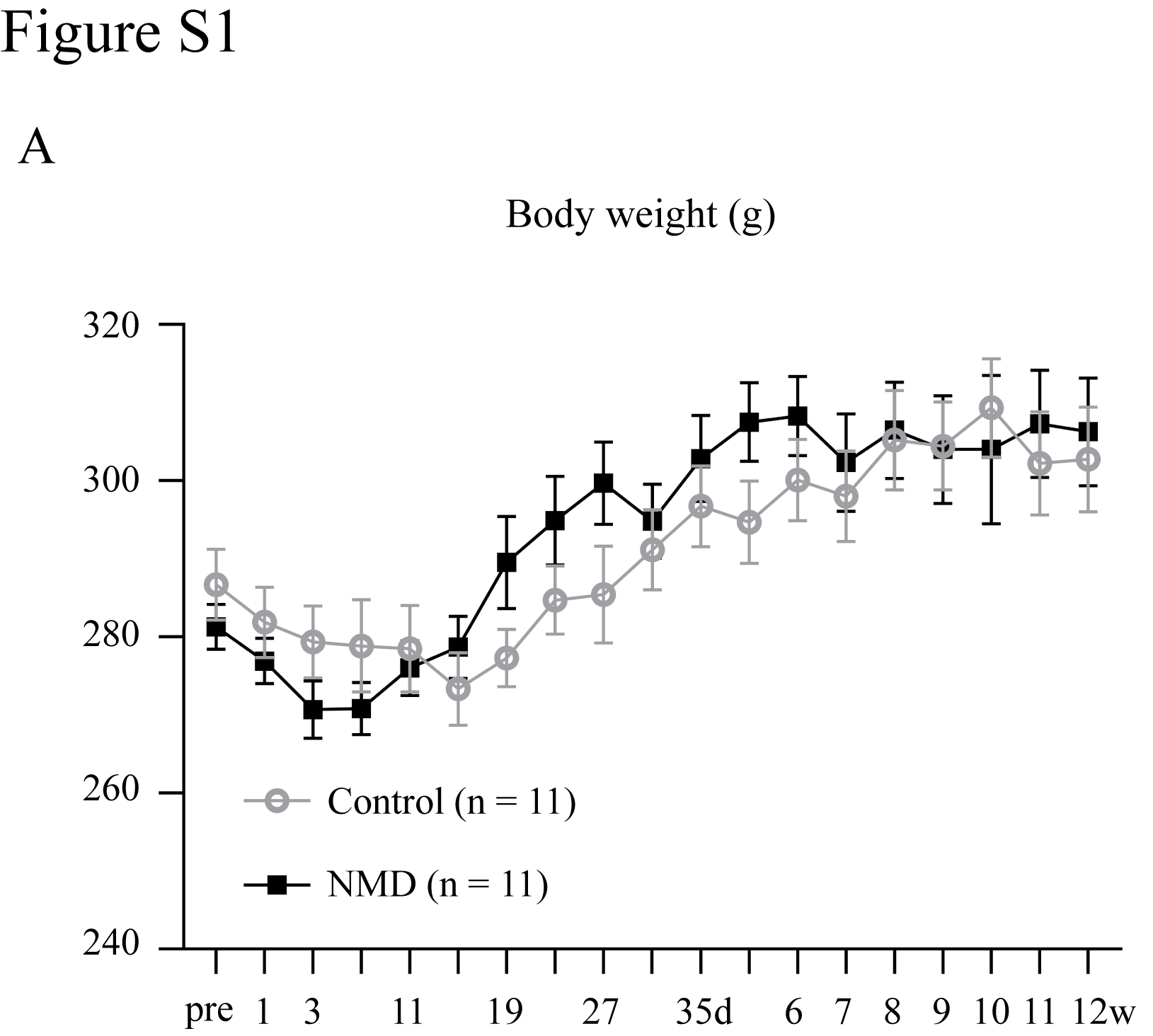

Supplement: Supplementary file 1 [file Image1.TIF]
